# Supplementary material for: Analysis of potential genetic biomarkers and molecular mechanism of smoking-related postmenopausal osteoporosis using weighted gene co-expression network analysis and machine learning
Source: PLoS One. 2021 Sep 23;16(9):e0257343. doi: 10.1371/journal.pone.0257343 (PMC8459994; doi:10.1371/journal.pone.0257343)
Supplement: Attachment — Submitted filename: Response to Reviewers.docx [file pone.0257343.s008.docx]

Response to Reviewers

Dear editors and reviewers:

Thank you for your letter and the reviewers’ comments on our manuscript entitled " Investigation of Potential Genetic Biomarkers and Molecular Mechanism of Smoking-related Postmenopausal Osteoporosis by Using WGCNA and Machine Learning" (ID：PONE-D-21-08513). Those comments are very helpful for revising and improving our paper, as well as the important guiding significance to other researches. We have studied the comments carefully and made corrections which we hope meet with approval. The main corrections are in the manuscript and the responses to the reviewers’ comments are as follows (the replies are highlighted in blue). In addition, we have added several important supporting information.

Replies to the reviewers’ comments:

Reviewer #1:

1. The author seems to have mixed analytical methods with subjective heuristics, which is concerning. For example, based on correlation analysis the author identifies the yellow module as showing a significant correlation between both smoking and osteoporosis. However, this identification seems subjective as the black module also seems to be significant. Futhermore, since this data is not RNAseq data but microarray data, which generaly profiles targeted transcripts, it is quite possible that the black module will also identify some relevant biomarkers. The author, therefore, needs to carefully, methodically and quantitatively justify the selection of the relevant module.

Response: According to your suggestion, we rewrote the “Correlation between gene modules and SRPO” in the “Methods and materials” and extended the reason why we chose blue module as the research object. In this study, we aimed to investigate potential genetic biomarkers and the underlying molecular mechanism of smoking-related postmenopausal osteoporosis. We collected the gene expression data of 20 postmenopausal female smokers (10 with high BMD and 10 with low BMD). Smoking was a common exposure for these people. The difference between these people was whether osteoporosis occurs or not, which may be related to the smoking‐induced genetic alterations. The genetic biomarkers of SRPO should differentiate SRPO patients and non-osteoporosis postmenopausal female smokers. So, we used WGCNA to identify the gene modules that highly correlated to the clinic trait (osteoporosis). After the construction of a weighted gene co-expression module network, module gene (ME) was used to evaluate the possible relationship of gene module with clinic trait. The Pearson correlation between ME and clinic trait was calculated to identify the module that was highly correlated to osteoporosis. The *t*-test was used to measure the significance of the Pearson correlation, and the module with a *P*-value less than 0.05 was considered to have a significant correlation to SRPO. Besides, the module significance (MS) of each module was calculated. The module with the highest absolute MS value was indicated to be significantly correlated to SRPO. As a result, the blue module (module–trait relationships = 0.88, *P*-value = 7e-07) was found to have the highest association with osteoporosis. And the blue module was found to have the highest MS value among all of the selected modules. Based on these results, we chose the blue module as the research object.

2. Following the above point the author should clarify how the correlation between smoking/osteoporosis phenotype and eigengene of the different modules is computed and how the significance is computed. Additionally, in the Results section the author mentions correlation between eigenvalue (instead of eigengene) and the phenotype. The authors should clarify this confusion.

Response: Thanks for the suggestion of the reviewer, we described the calculation method in detail in the “Methods and materials”. The WGCNA algorithm used module eigengene (ME) to evaluate the possible relationship of gene module with clinical trait. ME was defined as the major component computed by means of principal component analysis that recapitulates the manifestation of genes in a specific module into a single characteristic expression profile. The Pearson correlation between ME and clinical trait (osteoporosis) was calculated to identify the module that was highly correlated to SRPO. The *t*-test was used to measure the significance of the Pearson correlation, and the module with a *P*-value less than 0.05 was considered to have a significant correlation to SRPO. We are sorry to make a confusion of eigengene and eigenvalue. We have made corrections according to the Reviewer’s comments. In the module–trait relationships heatmap, the correlation between module eigengene and clinic trait was computed.

3. The author uses a recursive feature elimination approach. In the context of statistical regression, such stepwise approaches have been shown to be suboptimal. Why does the author think that this approach is a good option, especially given that many of the microarray genes might already be pre-selected and correlated. Clarification would be quite helpful.

Response: According to the suggestion of the reviewer, we rewrote the “Machine learning for feature selection” in the “Methods and materials” and extended the reason why we used the recursive feature elimination approach for the feature genes selection. In this study, we used support vector machine-recursive feature elimination (SVM-RFE), and random forest (RF) algorithm to select feature genes from hub genes that correlated to SRPO. The recursive feature elimination approach removed one feature with the smallest weight iteratively to a feature rank until all the features had been removed. In each iteration, the current SVM-RFE model was evaluated by the k-fold cross-validation. Finally, the classifier model with highest accuracy was constructed and the best variables were found. The support vector machine combined with recursive feature elimination algorithm had shown promising power in the analysis of the genomics, metabolomics, and proteomics. For instance, in the previous study, SVM-RFE had helped identifying biomarkers correlated with breast cancer prognosis (Li J, et.al Tumor Characterization in Breast Cancer Identifies Immune-Relevant Gene Signatures Associated with Prognosis. Front. Genet. 10:1119.). Besides, the main advantage of microarray chips is that they can simultaneously examine the expression of thousands of genes on a large scale and comprehensively. For example, through gene microarrays, genes that may be affected by a disease can be found in a short period of time and serve as biomarkers for early diagnosis. Therefore, we believed that the SVM-RFE machine learning algorithm is a good option for feature genes selection and we can use SVM-RFE algorithm to identify reliable biomarkers for SRPO.

4. What type of SVM did the author use: linear/non-linear, soft/hard margin, nu/C parameterization and why?

Response: We are very sorry for our neglect to describe the process of identification of feature genes using machine learning algorithms in detail. We have rewritten this part in the results according to the Reviewer’s suggestion. Firstly, an SVM-RFE classifier (Core: svmliner; Cross: 10-fold cross-validation; soft-margin; tuning parameter C = 1) was established based on the 113 hub genes. The data of control group and SRPO group was randomly divided into 10 equal portions (training set: 9, test set: 1). During each of the ten iterations, the SVM-RFE algorithm was performed on the training set to train the classifier with the selected features. Next, the trained classifier was applied to the test set for prediction. Then, the predictions from all 10 iterations were combined to evaluate the accuracy of the classifier. By the SVM-RFE algorithm, we validated a set of 8 feature genes (Fig. 6A). In our SVM-RFE model, the tuning parameter C (also known as Cost) was set as default (C=1). And we used the soft-margin in our SVM-RFE for the reason that soft-margin SVM could choose decision boundary that has non-zero training error even if dataset is linearly separable, and is less likely to overfit. Besides, during the feature selection, we tried several types of SVM models，including linear, radial, sigmoid. Finally, the SVM-RFE classifier (Core: svmliner; Cross: 10-fold cross-validation) with these 8 feature genes revealed the highest predict accuracy, and this classifier was selected by the SVM-RFE algorithm. The prediction result based on the selected SVM-RFE classifier has been provided as a supplementary file.

5. The author should clarify their validation approach: what was the training set, what was the testing set, and how did the control set relate to them.

Response: The comments are very helpful for revising and improving our paper, and we have made correction according to the Reviewer’s comments. We rewrote the “Data validation” and described this section as “Evaluation of the diagnostic efficiency”. After integrating the results of feature selection from SVM-RFE and RF, we obtained six feature genes highly correlated to SRPO. The ability of feature genes to differentiate between SRPO patients and non-osteoporosis postmenopausal smokers was evaluated by gene expression and ROC curve analyses. The predictive efficiency was measured in the control group (ten samples from postmenopausal smokers with high BMD) and the SRPO group (ten samples from postmenopausal smokers with low BMD). As smoking was the common exposure for these people, the occurrence of SRPO between them may be highly related to the smoking‐induced genetic alterations. The biomarkers of SRPO should distinguish SRPO patients and non-osteoporosis postmenopausal female smokers precisely. As a result, the expression pattern of selected feature genes was significantly differentiated between the two groups. Moreover, these feature genes reached great diagnosis value with an AUC＞0.9, respectively. Furthermore, we committed certain limitations of this study in the discussion. There was little research on genomic variation in SRPO for now. To our best knowledge, this study is the first to investigate potential genetic biomarkers of SRPO. However, functional validation and external validation of these biomarkers were lacked in this study. The reliability of this study still needs to be verified by further experiments.

6. It remains unclear to me as to why the author has generated ROCs and related AUC only for individual gene expressions. The performance is quite poor, and if one looks at the shape of the ROC, the sensitivity and specificity are not good either. It might be better to generate ROC curves for gene expression combinations.

Response: We are very sorry for our neglect to explain the purpose of the ROC analysis for individual gene expressions of the feature genes. We rewrote the “Data validation” and described this section as “Evaluation of the diagnostic efficiency” and we extended the reason why we performed ROC analysis for the feature genes in this section. In order to identify if the feature genes influence the SRPO diagnosis independently, we performed receiver operating characteristic (ROC) curve analysis for the selected feature genes on individual gene expression. As a result, the six feature genes reached great diagnosis value with an AUC＞0.9, respectively. Moreover, the shape of the ROCs indicated great sensitivity and specificity of these feature genes in SRPO diagnosis. It might be a good idea to generate ROC analysis for gene expression combination of all the feature genes. But the above ROC results of individual feature gene expressions have proven that the feature genes can influence the SRPO diagnosis independently.

7. In the abstract the author has mentioned the "yellow module" as the clinically significant module. This seems to be a highly unusual way to present ones results. Nobody reading the abstract will understand what a yellow module is. The author needs to provide a better description.

Response: We are sorry that our English writing level has troubled the reviewers. We have submitted our manuscript to TOPEDIT (<https://www.topeditsci.com/>) for polishing as suggested by the reviewers. Correction have been made as follow. Eight highly conserved modules were detected in the WGCNA network, and the genes in the module that was strongly correlated with SRPO were used for constructing the PPI network.

8. The writing needs to be improved. For example, 'According to recently scientific discoveries, ...', 'With the rapid development of high-throughput microarray technologies, identification of meaningful genomic variations and investigating biological mechanisms have contributed great effort ....', etc.

Response: We apologize for the language problems in the original manuscript. We have submitted our manuscript to TOPEDIT (<https://www.topeditsci.com/>) for polishing as suggested by the reviewers.

Reviewer #2:

Major

1. Identification of significant modules and analysis of module-trait relationship – It was not clear whether proper multiple-testing correction has been applied. This appeared to be critical as the significance of the two modules nominated in the Module-Osteoporosis analysis is marginal (Yellow p=0.03, Green p=0.02). And the significance of the Yellow in Module-Smoking relationship will also become marginal if Bonferroni is applied to correct 11 tests.

Response: According to your suggestion, we rewrote the “Correlation between gene modules and SRPO” in the “Methods and materials”. In the revised paper, we aimed to investigate potential genetic biomarkers and the underlying molecular mechanism of smoking-related postmenopausal osteoporosis. The difference between the SRPO and control groups was whether osteoporosis occurs or not, which may be related to the smoking‐induced genetic alterations. The genetic biomarkers of SRPO should differentiate SRPO patients and non-osteoporosis postmenopausal female smokers. Thus, we used WGCNA to identify the gene modules that highly correlated to the clinical trait (osteoporosis). In WGCNA algorithm, the number of statistical tests is greatly reduced by clustering the genes into modules and then making statistical analysis. The association between thousands of genes and phenotypes is transformed into that between several gene sets and phenotypes, so as to avoid the problem of multiple hypothesis testing and correction. This processing method strengthens the strong correlation and weakens the weak or negative correlation, making the correlation value more consistent with the characteristics of scale-free network and more biologically meaningful. Taken together, the proper multiple-testing correction has been applied in the WGCNA algorithm. As a result, the blue module (module–trait relationships = 0.88, *P*-value = 7e-07) was found to have the highest association with osteoporosis. And the blue module was found to have the highest MS value among all of the selected modules. Based on these results, we chose blue module as the research object.

2. Construction of PPI network – The network and sub-network built in this paper seemed to be very dense. This was likely resulted from the authors including all sorts of protein linkages. Restricting to only “experimentally determined interactions” may better clarify the biological meaning of the network and the subsequent results. In particular, the inclusion of “gene neighborhood interactions”, “text-mining interactions”, and “gene co-occurrence interactions” lacks biological context.

Response: We are very grateful to the reviewer for this helpful suggestion. In order to investigate the biological interaction among the genes in the selected modules from WGCNA, we performed a protein-protein interaction (PPI) network analysis for these genes using the STRING database. After removing the disconnected nodes, there were 998 nodes and 10940 edges in the constructed PPI network. Then, we used the CytoNCA plugin for Cytoscape to perform a topological network analysis in the PPI network. We set the twice median DC value as a threshold to screen significant nodes in the PPI network and generate a subnetwork. Furthermore, nodes where both BC and CC value were greater than the median in the subnetwork were identified as a new core network containing hub genes. Finally, the core network containing 113 hub genes (nodes) and 1831 edges was displayed. The identified hub genes were subjected to enrichment analysis to investigate their biological meaning.

3. Function and pathway enrichment analysis – 1) Statistical significance again was not properly defined here. While gene set enrichment tools usually provide adjusted p-values (or q-values), the authors stated that “The p-value < 0.05 was considered to indicate a significant difference for GO terms and KEGG pathways”. 2) Statistical significance (Adjusted P-value or Odds) would be preferred in the figure, instead of “gene number”. 3) It is of little use to list all the enriched terms in the main text, instead, the authors should investigate further and explain the biological meaning of these terms and more importantly, how they are relevant to the phenotype SRPO. This is essential and is part of be the meat of this paper, as the authors claimed that this work could provide novel insights into the molecular mechanism of SRPO.

Response: We have made corrections according to the reviewer’s comments. 1) In “Function and pathway enrichment analysis”, a Benjamini–Hochberg adjusted *P*-value < 0.05 was considered to indicate a significantly enriched GO terms and KEGG pathways. 2) In Fig.5, the “Gene Ratio” was used for statistical significance display. 3) The results of function and pathway enrichment analysis of hub genes indicated that hub genes were mainly concentrated in the regulation of RNA transcription and translation, regulation of cell cycle, ATPase activity, HIF-1 signaling pathway, and NF-kappa B signaling pathway, These GO terms and KEGG pathways seem to have closely association with the development of SRPO. We extended the biological meaning of these terms and their association with SRPO in the discussion.

4. Identification of feature genes using machine learning method – It was not sufficiently justified why the specific two ML methods were chosen. Also, how the recursive feature elimination analysis was performed and how it can benefit the model was not clearly explained. Plus, while prioritizing the genes by their overlaps seems justifiable, more detailed comparison of the two sets of genes is desired to give a more comprehensive view of the results (e.g., Are those non-overlapping genes also functionally relevant? Does one gene set appear to be more relevant than the other? Why RF gave many more genes than SVM – is it more powerful or less accurate? Would it be reasonable to use the union instead of intersection to implicate more genes?).

Response: According to the Reviewer’s suggestion, we extended the reason why we chose SVM-RFE and random forest algorithm for feature genes selection and how these two machine learning algorithms were performed in the “Methods and materials”. In this study, feature genes associated with SRPO were selected using SVM-RFE and RF. SVM-RFE was an efficient feature selection algorithm and had shown promising power in the analysis of the genomics, metabolomics], proteomics, etc. For instance, in the previous study, SVM-RFE had helped identifying biomarkers correlated with breast cancer prognosis (Li J, et.al Tumor Characterization in Breast Cancer Identifies Immune-Relevant Gene Signatures Associated with Prognosis. Front. Genet. 10:1119.). During the performance, the recursive feature elimination approach removed one feature with the smallest weight iteratively to a feature rank until all the features had been removed. In each iteration, the current SVM-RFE model was evaluated by the k-fold cross-validation. Finally, the classifier model with highest accuracy was constructed and the best variables were found. RF had also been widely used for detecting biomarkers in many diseases. The RF algorithm used the variables to construct numerous decision trees and generated the most accurate classes of variables to individual trees. Referring to other similar studies, we used SVM-RFE and RF machine learning algorithms to select feature genes of SRPO in this study. Moreover, we performed a combination strategy to minimize the possibility of losing important features through incorporating genes from two machine learning algorithms. Overlapping the feature genes selected by different machine learning algorithms to identify the most important feature genes is a very common processing method in many previous studies. SVM-RFE and RF machine learning algorithms screened characteristic variables based on different algorithm theories, so the number of feature genes screened out by these two machine learning algorithms were different. We believed that the common feature genes selected by both machine learning algorithms are important genes that are closely related to SRPO. In the following analysis, we found that the six overlapped feature genes show great diagnostic efficiency for SRPO, which indicated the reliability of our research methods. We hope the above explanation can address the reviewer’s comment.

5. Data validation – 1) The selection of control group: “10 samples of postmenopausal non-smoking females with high BMD” was used, while alternatively, the other group “10 samples of postmenopausal non-smoking females with low BMD” can be used to better dissect the relationship between the implicated genes and “smoking-related” postmenopausal osteoporosis (rather than the general postmenopausal osteoporosis). 2) Multiple-testing correction is expected for the gene expression analysis; if Bonferroni, genes ATP5G1 and RPL26L1 will not surpass the threshold. 3) Description and discussion about the functional significance of the implicated genes are expected to follow the results here. The authors had lengthy paragraphs on this in the Discussion section, which should be moved up, expanded in depth, while rephrased more concisely.

Response: 1) We are very grateful to the reviewer for the helpful comment and we made correction according to the reviewer’s suggestion. In this study, we aimed to investigate potential genetic biomarkers and the underlying molecular mechanism of smoking-related postmenopausal osteoporosis. We collected the gene expression data of 20 postmenopausal female smokers (10 with high BMD and 10 with low BMD). Smoking was the common exposure for these people. The difference between these people was whether osteoporosis occurs or not, which may be related to the smoking‐induced genetic alterations. The genetic biomarkers of SRPO should differentiate SRPO patients and non-osteoporosis postmenopausal female smokers. So, we selected 10 sample of postmenopausal female smokers with high BMD (non-osteoporosis) as the control group, 10 sample of postmenopausal female smokers with low BMD (osteoporosis) as the SRPO group in this study. 2) According to the suggestion of reviewer, we performed multiple-testing correction for the gene expression analysis. In the gene expression analysis of the selected feature genes, the *t*-test with multiple-testing correction (Benjamini–Hochberg) was used to test for significant differences between the two groups. As a result, the six feature genes, HNRNPC (*p*=0.0031), PFDN2 (*p*=0.0013), PSMC5 (*p*=0.0371), RPS16 (*p*=0.0202), TCEB2 (*p*=0.0073), and UBE2V2 (*p*=4.07e-05) was found statistically significant differentiated between the two groups. 3) According to the reviewer’s suggestion, we rephrased the description and discussion about the functional significance of the implicated genes in the discussion. Through extensive reading of relevant literatures, we explored the biological association between these feature genes and smoking-related postmenopausal osteoporosis, hoping to discover the potential mechanism of these genes in the pathogenesis of SRPO.

Minor

1. Accurate references need to be cited when linking the implicated genes/pathways to SRPO if the relevant conclusions are not drawn by this paper (e.g., “It has been suggested that aging and increasing in reactive oxygen species (ROS) may be the proximal culprits for osteoporosis”, “ROS can influence the generation and survival of osteoclasts and osteoblasts” …).

Response: We are very grateful to the reviewer for the comment and we have made corrections to this section. The accurate references were cited in the discussion to link the implicated genes/pathways to SRPO.

2. Grammar needs to be revised (small errors like "Osteoporosis is one of the most common systemic skeletal disorder" occurs occasionally).

Response: We apologize for the language problems in the original manuscript. We have submitted our manuscript to TOPEDIT (<https://www.topeditsci.com/>) for polishing as suggested by the reviewers.

3. Language needs to be polished into a more scientific fashion (for example, in “… performed a heatmap and bar graph of ...” and “…scatterplots of GS vs. MM of module yellow of the two phenotypes were performed …”, specific analyses/statistical tests should be described rather than the types of graphs).

Response: We are sorry that our English writing level has troubled the reviewers. We have submitted our manuscript to TOPEDIT (<https://www.topeditsci.com/>) for polishing as suggested by the reviewers.

4. Data availability – key results should be complied as supplementary files for others to use (e.g., module information, PPI network, pathways, and relevant statistical results, etc.).

Response: We are very grateful to the reviewer for this helpful suggestion. We have provided the original gene expression matrix, module information, PPI network, results of GO and KEGG enrichment analysis, and results of machine learning as supplementary files, based on the reviewers' recommendations.
