# Supplementary material for: Analysis of potential genetic biomarkers and molecular mechanism of smoking-related postmenopausal osteoporosis using weighted gene co-expression network analysis and machine learning
Source: PLoS One. 2021 Sep 23;16(9):e0257343. doi: 10.1371/journal.pone.0257343 (PMC8459994; doi:10.1371/journal.pone.0257343)
Supplement: Attachment — Submitted filename: Response to Reviewers.docx [file pone.0257343.s009.docx]

Response to Reviewers

Dear editors and reviewers:

Thank you very much for your attention and the reviewers’ evaluation and comments on our manuscript entitled "Analysis of potential genetic biomarkers and molecular mechanism of smoking-related postmenopausal osteoporosis using weighted gene co-expression network analysis and machine learning" (ID：PONE-D-21-08513R1).

We have revised the manuscript according to your kind advices and reviewers’ detailed suggestions. Enclosed please find the responses to the reviewers. We sincerely hope this manuscript will be finally acceptable to be published on PLOS ONE.

Thank you very much for all your help and looking forward to hearing from you soon.

Best regards

Sincerely yours

Replies to the reviewers’ comments:

Reviewer #1:

The authors have adequately addressed my comments. In particular, I was glad to see that in their Discussion the authors have moderated the significance of their results with the limitations inherent in this initial study.

Response: We are grateful to Reviewer #1 for his/her effort reviewing our manuscript and his/her positive feedback. The summary of our work as written by this reviewer is precise. Thanks again for your support of our manuscript.

Reviewer #2:

The manuscript has been considerably improved. The authors addressed all technical comments well; the results are now scientifically sound and worth to be published.

One last improvement needed is the writing / organization of the paper. The results section reads too thin to deliver the message fully. The major issue appears to be lacking of relevant discussion/interpretation following specific results. For example, result #2 "Construction of the PPI network" appears insufficient to be an independent section (it's more like a paragraph of Method). An easy fix could be combining it with result #3. Similarly, #5 "Diagnostic efficiency of feature genes" is also too thin - further interpretation is expected, possibly merge with result #4. On the other hand, the authors did a decent amount of work on explaining the specific nominated genes in the Discussion section. I'd suggest move these discussions to the Result section to alleviate the scarcity and better deliver the scientific implication of the analyses.

Response: We appreciate Reviewer #2 for his/her effort to review our manuscript, and his/her positive feedback. The reviewer gives an accurate summary of our work and brings forward constructive questions. We have addressed them below. According to Reviewers’ suggestion, we combined result #2 with #3. Besides, we agreed with the comments that result #5 "Diagnostic efficiency of feature genes" is thin, and we have moved the explanation of the feature genes in the Discussion to result #5 according to the suggestion. We also extended the relationship of the identified feature genes in our study and smoking-related postmenopausal osteoporosis in the Discussion section. We sincerely hope our responses can address Reviewers’ comments.
